# Online self management training for patients with food allergy

**DOI:** 10.1186/2045-7022-3-S3-P130

**Published:** 2013-07-25

**Authors:** H Van Os-Medendorp, A Michelsen, M Pronk-Kleinjan, A Knulst

**Affiliations:** 1 Dermatology & Allergology, UMC Utrecht, Utrecht, the Netherlands

## Background

Patients with food allergy generally have a strong need for information on their disease and disease management. Their daily life is full of uncertainties. Therefore an online self management training “Living with food allergy” has been developed for patients with a physician’s diagnosed food allergy to increase patient’s self management. The training was developed by a multidisciplinary team of a dietician, dermatologist, nurse and researcher in collaboration with patients’ organizations: the Dutch Food Allergy Foundation and the Netherlands Anaphylaxis Network; The Netherlands Nutrition Centre Foundation and ICT experts. Financial support for development of the training was obtained from the Innovation Fund of insurance companies. The training consists of information, video’s, patient’s stories and exercises about food allergy, diagnostics, coping with unexpected reactions, diet and consequences of food allergy in daily life.

## Methods

A pilot study to evaluate use, usability and acceptability of the online training was carried out under adult patients, GP’s and medical specialists, according to the technology acceptance model. After the pilot, the training has been offered in daily practice, in addition to the care of general practitioners, dieticians or medical specialists.

## Results

Results of the pilot study showed that the online training was well accepted by patients and health care providers. The online training was evaluated as useful in addition to the usual care of the patients and easy to use. After the pilot phase, more than hundred adult patients were referred to the training, with various allergies as peanuts, nuts, milk, fruits or vegetables. Characteristics of the patients and experiences with use of the training in daily practice will be presented at the congress.

## Conclusion

The online self management training “Living with food allergy” is a useful addition in the treatment of patients with food allergy and available for all food allergic patients in the Netherlands.

## Disclosure of interest

None declared.

